# Forgotten Variables in Gerontological Evaluation of Religiosity and Spirituality

**DOI:** 10.1093/geroni/igaa057.1991

**Published:** 2020-12-16

**Authors:** Nadia Firdausya, Alex Bishop, Tanya Finchum

**Affiliations:** 1 Oklahoma State University, stillwater, Oklahoma, United States; 2 Oklahoma State University, Stillwater, Oklahoma, United States; 3 Oklahoma Oral History Research Program; Oklahoma State University, Stillwater, Oklahoma, United States

## Abstract

Data for this study originated from the Oklahoma 100 Life Project. The purpose of this investigation was to explore oral history storytelling surrounding centenarian religious practices in childhood. Thematic content analysis of oral narratives revealed three prominent themes surrounding the transmission, accessibility, and socialization of religion. Reflection upon the intergenerational transmission of religion, one centenarian recounted if her mother didn’t go to church then, “the kids didn’t go.” Another centenarian noted the influence of rural opportunity structure stating, “Out there in the cotton patch, we didn’t have no church.” Finally, one centenarian participant highlighted value of social value of church during childhood by exclaiming, “We went to church all the time. . .we didn’t know what it was to stay home from church.” Findings will be used to further highlight how assessment of early life experiences is essential to gaining a fuller understanding of religiosity in human longevity.

